#  “Can she hear me?”—Questions of experience and suffering in cognitive motor dissociation

**DOI:** 10.3389/fneur.2026.1858354

**Published:** 2026-05-28

**Authors:** Alice Hawkins

**Affiliations:** Department of Neuropalliative Care, Cedars-Sinai Medical Center, Los Angeles, CA, United States

**Keywords:** cognitive motor dissociation, covert consciousness, disorders of consciousness, neuropalliative care, neurotechnology

## Abstract

Care for patients with disorders of consciousness (DoC) has been transformed by advanced neurotechnologies capable of detecting cognitive-motor dissociation (CMD)—volitional brain activity in patients who appear unresponsive on bedside examination. Recent evidence suggests that up to one in four patients diagnosed with unresponsive wakefulness syndrome may harbor covert consciousness, and that CMD is associated with significantly improved functional outcomes. These findings have challenged a longstanding therapeutic nihilism in the care of patients with DoC, and professional societies now recommend routine CMD assessment to inform advance care planning. However, the limitations of these technologies warrant careful consideration. This article examines the ethical implications of CMD detection from a neuropalliative care perspective. It argues that suffering in patients with DoC extends beyond nociception to encompass existential dimensions that are often overlooked, and that the presumption of consciousness should guide clinical care regardless of imaging findings. Furthermore, it proposes that advanced neurotechnologies should be deployed not only as diagnostic and prognostic tools, but as therapeutic instruments for communication, connection, and quality of life—benefits that hold value independent of functional recovery. As technological capabilities in consciousness detection advance, the ethical obligation to attend to the present experience of these patients deepens correspondingly.

## Introduction

As a neuropalliative specialist, I regularly care for patients with disorders of consciousness in critical care settings, and counsel their families through decisions about their care. Some of the most common—and yet challenging—questions I hear from family members concern the nature of their loved ones' subjective experience. Are they able to hear us when we are in the room? Do they feel pain? Are they suffering? It is already difficult to prognosticate accurately about a patient's future, and yet somehow I find myself even more humbled when confronted with questions of their current state of being as they lie in their hospital bed before me.

Care for patients with disorders of consciousness (DoC) has evolved in recent years, owing largely to advanced neurotechnologies that aid not only in prognostication but also in the assessment of possible covert consciousness undetectable on physical examination, known as cognitive-motor dissociation, or CMD. The discovery of patients with DoC and CMD carries important implications not only for their care and advance care planning, but for care of all patients with DoC. In this article, I will explore ethical and palliative considerations that are unmasked by these new advances.

## Definitions of disorders of consciousness

Disorders of consciousness (DoC) are clinical syndromes that can arise from various mechanisms, such as severe acute brain injury (SABI), cerebrovascular events, or neurodegenerative disease. DoC are defined largely by alterations in two cardinal attributes—awareness and arousal. Patients in coma demonstrate neither awareness nor arousal (wakefulness). The syndrome of wakefulness in the absence of awareness was formerly referred to as vegetative state (VS). However, many now prefer the less pejorative term “unresponsive wakefulness syndrome” (UWS) (1). Patients in UWS demonstrate no awareness of their environment, but show evidence of arousal through spontaneous eye opening, yawning, and sleep/wake cycles on electroencephalogram (EEG). They may additionally demonstrate other affective behaviors, such as grimacing or crying, that are thought by many to be subcortical reflexes rather than a conscious response to pain—a point that bears some nuance and will be discussed further later in this article. Some patients with UWS emerge into a minimally conscious state (MCS). Patients in MCS demonstrate arousal and show some evidence of awareness of their environment, ranging from visual tracking to following commands or gesturing to simple yes/no questions. What is termed “awareness” in the realm of medical definitions of consciousness can also be described as “subjectivity” or “subjective experience” in more philosophical terms. Philosophers in phenomenological thought in particular, such as Edmund Husserl, Martin Heidegger, and Jean-Paul Sartre, view consciousness as not merely subjective experience *of oneself* , but perception of and direction toward the outside world in some way—a feature analogous to “awareness” in the neurological sense (2).

## Cognitive-motor dissociation: evidence and clinical significance

Historically, the assessment of awareness in DoC has relied heavily on the bedside neurological examination. Findings such as visual pursuit, localizing to stimuli, or command-following that may be seen in patients with MCS suggest that in these patients, higher-order cognitive processing is intact, whereas in coma or UWS, it is absent. However, advanced imaging techniques, such as task-based functional magnetic resonance imaging (fMRI) or EEG, have demonstrated that some patients with UWS or MCS show evidence of volitional brain activity in response to specific cognitive tasks such as command-following - despite lacking physical movement that has traditionally served as our only reliable proxy measure for these cognitive functions. This dissociation between awareness and observable motor responses is known as cognitive-motor dissociation, or CMD (3). This is not a rare phenomenon either—according to recent data, up to 1 in 4 patients who are clinically unresponsive on bedside examination and diagnosed with UWS may demonstrate willful modulation of brain activity in response to command (4, 5).

Evidence of CMD—and of disparities between bedside examination and advanced imaging—has been accumulating for over two decades. As early as 2005, Schiff et al. (6) described passive language processing in patients diagnosed with MCS via cortical network activation on fMRI that was similar to healthy controls in response to personal narratives read by family members. This activation was absent in response to meaningless linguistic stimuli. Shortly thereafter, Owen et al. (7) published a landmark case report in 2006 describing a patient diagnosed with vegetative state who activated cortical areas indistinguishable from healthy volunteers when asked to imagine playing tennis, or move through the rooms of her house. In 2010, Monti et al. (8) demonstrated in their cohort of ICU patients that close to 10% of patients with UWS may be able to willfully modulate brain activity on fMRI in response to command, using a visualization paradigm similar to that of Owen et al. Additionally, Cruse et al. (9) demonstrated that patients diagnosed with vegetative state could generate appropriate EEG responses to command via imagined motor activation of their right hand or toes.

Beyond their academic significance, findings of CMD have meaningfully influenced prognostication and clinical outcomes. Several studies have demonstrated that patients with acute brain injury diagnosed with UWS and CMD have improved functional outcomes compared to patients with UWS who do not show evidence of CMD (10–12). In the cohort studied by Claassen et al. (10), patients with UWS and CMD were over four times more likely than patients with UWS without CMD to achieve functional independence—defined as not requiring a caregiver for at least 8 h daily—at 12 months post-injury. This level of recovery is not merely statistically significant. It is profoundly important to surrogate decision makers when considering continuation of life-sustaining treatments after injury, and meaningfully impacts patient quality of life. Given the promising nature of this data, many professional societies now recommend routinely assessing for CMD in ICU and long-term care settings to aid in advance care planning and medical decision making (13, 14).

## Therapeutic nihilism vs. cautious optimism

For good reason, the discovery of CMD, and its positive impact on prognosis, has been a wellspring of optimism in the treatment of patients with DoC. This is in stark contrast to a therapeutic nihilism that had pervaded the care of patients with DoC since the landmark New Jersey Supreme Court case of Karen Ann Quinlan in 1976, who was diagnosed with vegetative state after anoxic brain injury. Due to concerns of suffering, her parents requested to disconnect her from her mechanical ventilator and allow a natural death, a request contested by the county prosecutor, who threatened homicide charges on the hospital. Expert testimony from Dr. Fred Plum, a court-appointed neurologist, highlighting her loss of a “cognitive sapient” state, provided the moral underpinning for the court's decision to allow withdrawal of life-sustaining treatment. This case, as well as several others, has propelled the right-to-die movement in the United States, in both the medical community and the public sphere (15). Joseph Fins, a long-time advocate for dignity and optimism in caring for patients with DoC, has described this trend as equating disorders of consciousness with medical futility, which has resulted in a sense of therapeutic pessimism in the care of these patients (16).

## Limitations of advanced neurotechnologies

It is important to be mindful of the limitations of routine testing for CMD and its implications, lest the pendulum swing too far from pessimism to uncritical optimism. In the Monti et al. (8) study, using fMRI to elicit evidence of consciousness also revealed the potential for false-negative results—patients who showed no reproducible fMRI response despite having been clinically diagnosed with MCS on repeated bedside assessments. In Bardin et al. (17) study of an fMRI imagined motor imagery paradigm to detect command-following in patients with severe acute brain injury, one of the three subjects who failed to demonstrate command-following later emerged from MCS and reported that she had understood the task and attempted to perform it.

These cases highlight real issues with the sensitivity of these advanced imaging modalities, because insufficient sensitivity risks introducing incomplete or inaccurate information into diagnostic and prognostic assessments, and ultimately into clinical decision-making. Fascinating as the potential for CMD is, it may inadvertently reinforce a neuro-essentialist framework—one that reduces mental phenomena to discrete physiological processes, at the expense of broader conceptions of mind, personhood, or consciousness (18). In the Monti et al. study (8), one patient diagnosed with UWS did show evidence of CMD on fMRI, however on further more rigorous bedside testing met criteria for MCS. This shows that while there is a potential advantage in this technology identifying behaviors that may be missed on bedside assessments, one should exercise caution to avoid supplanting technology in place of careful bedside examination given their equally demonstrated false-negative rates.

Not only does substituting advanced imaging modalities for clinical rigor at the bedside lead to misdiagnosis that may be easily avoidable (19), it risks encouraging clinicians and families to deny personhood to these vulnerable patients when imaging fails to demonstrate sufficient evidence of consciousness. Given these potential pitfalls, many clinicians, ethicists, and researchers advocate for presuming consciousness in patients that are unresponsive (5, 20, 21), to avoid the morally injurious risk of denying these patients their fundamental human rights, or underacknowledging their potential pain and suffering. This is in contrast to the guidance of The Multi-Society Task Force on PVS (persistent vegetative state) in 1994 (22) that stated that patients with PVS “are unaware and insensate and therefore lack the cerebral cortical capacity to be conscious of pain.”

Consciousness and unconsciousness do not exist on a pure binary scale, as there is a wide spectrum of experiences that can be considered in the realm of consciousness, such as pain, emotion, communication, and cognition or executive function. Therefore, to demarcate some patients as fundamentally unable to experience pain based on a binary clinical diagnosis, in the field neuroscience that many argue is still in its infancy, seems to be an overstep, as argued by Birch (23). This is critically important, as there high rates of misdiagnosis of VS/UWS vs. MCS (24). Moreover, the attribution of consciousness and therefore personhood to a patient with DoC may potentially facilitate their recovery (21). This is because several studies have demonstrated that patients with UWS or MCS that are presented with personally relevant stimuli, either biographical narratives or the voices of their loved ones, have increased cortical activation and, at times, greater functional recovery than patients without this personalized stimulation. Given these practical and moral concerns, and the demonstrated limitations in assessments of distinguishing levels of DoC using bedside assessment (and arguably the advanced neurotechnologies used to detect CMD), it is imperative to exercise what Matthew Braddock terms “precautionary personhood.” This argues that in the face of uncertainty about a patient's level of awareness or consciousness, one should default to the assumption that they are conscious (25), because it would be morally more problematic to deny a conscious person their personhood, than it would be to ascribe personhood to an unconscious person. The emerging evidence of CMD arguably serves just as much to deepen uncertainty about patients who cannot reliably demonstrate consciousness, as it does to resolve uncertainty for patients who can.

## Pain, suffering, and existential dimensions of DoC

While the discovery of CMD has engendered a sense of optimism about recovery for patients with disorders of consciousness, it conversely has also unveiled a greater capacity for suffering than was previously presumed in these patients. Much like by the 1994 PVS Task Force previously mentioned, it has been postulated by others that comatose patients or patients with UWS lack the capacity to experience pain (26), while patients with MCS do show some evidence of higher cortical associative network activation in response to noxious stimuli, suggesting some capacity for pain processing (27). Others still, like Panksepp et al. (28), propose that this view is overly cortico-centric, and cite that many subcortical structures are involved in affective experiences like nociception, emotion, and biostatic processes like hunger and thirst. Whether there is cortical or conscious “awareness” of these affective experiences should not invalidate their very existence and reality for patients with DoC.

Given the rates of misdiagnosis that we have already discussed, and an overall lack of consensus about what neural structures and networks are sufficient to define “pain,” an inherently subjective experience, many authors have argued that empiric analgesia or palliative sedation should be considered morally imperative for patients with disorders of consciousness, so as to avoid under-treatment of pain (26, 29). The decision to pursue palliative pain management for these patients, however, needs to be carefully weighed against potentially conflicting goals of neurorehabilitation and preserving alertness, and should be readdressed over time (30). Even if one assumes that higher cortical processing is necessary for the experience of pain, the ambiguity about an individual's conscious experience that is brought about by a negative assessment for CMD remains due to the sensitivity limitations that have been mentioned, and therefore prophylactic or empiric pain management should be strongly considered for all patients with DoC, not just those with UWS+CMD or MCS.

A deeper—and perhaps more troubling—source of suffering may be the capacity for existential distress. Focusing on nociception and pain as the primary proxy measure of suffering is certainly more tangible and feasible in clinical settings, but this is a medicalized lens that neglects broader existential dimensions of suffering. After all, some of the most influential works on human suffering have located its origins in more abstract phenomena—desire, the illusion of freedom, and the burden of consciousness itself. Philosophers in the pessimist tradition have gone so far as to equate consciousness itself with suffering. These include Arthur Schopenhauer, who drew on Buddhist conceptions of suffering as rooted in desire (31), to more recent thinkers such as Peter Wessel Zapffe, who regarded human consciousness itself as a “tragedy,” in which human intellect becomes a source of anguish through an insatiable search for meaning (32). For Zapffe, our main coping mechanisms are the ability to engage in distraction from, and suppression of, these troubling thoughts—mechanisms fundamentally unavailable to patients with either DoC or CMD. These are not merely abstract thought experiments—quality of life (QoL) in the realm of healthcare involves complex emotional, spiritual, social, and physical aspects, the ability to pursue one's goals, and engage in activities that can provide fulfillment (33–35). These more metaphysical aspects of QoL are often considered to be more important than physical complaints, such as pain (36). It would thus be a gross oversimplification to look at the suffering of patients with coma or UWS purely in physical terms. One should consider all patients with DoC as having the potential for existential distress, because they still retain the possibility of affective emotional experiences such as fear or anxiety, and may even have some level of cognition intact that is not able to be elicited on bedside examination or with advanced neuroimaging. With the advent of CMD-related technologies, some patients with DoC may be identified that may have the potential to have agency in their lives, and have some hope of a degree of self-actualization that has been inaccessible until this point in time. But regardless of a patient's ability to demonstrate CMD, they can still benefit from other emotional needs being met, such as being in comfortable environments, enjoying music, and listening to loved ones even if higher cognitive tasks are unavailable to them (37, 38).

## Decision-making and meaningful use of neurotechnologies

This brings the discussion to a critical question: how should medical decisions be made for—and potentially by—patients with CMD? Without reliable communication, decision-making authority defaults to surrogate decision-makers, who are able to speak to the patient's previously stated wishes, or if none have been voiced, employ substituted judgment by applying the patient's personal values and preferences to the decision at hand (26). This has historically been the primary mode of decision-making for patients with DoC. One recent study showed that the incorporation of CMD detection into neuroprognostication was perceived to be helpful by surrogate decision makers, particularly when this finding is associated with long-term recovery (39). Prognostic considerations aside, surrogate decision makers are likely to have questions about the nature of consciousness that is revealed by CMD (e.g., whether loved ones have a rich subjective experience of their surroundings, or if they are able to feel pain), and there is a lack of professional guidance for how to communicate with surrogates about these findings. Some surrogates may experience distress at the possibility that their loved one is conscious and suffering, while others may find their present time with their loved ones more meaningful if they are presumed to be conscious. Clinicians should therefore be prepared for a spectrum of responses to the possibility of CMD.

Another important question is whether patients with CMD may be able to make medical decisions for themselves. As Istace (40) highlights in a review of decision-making for patients with CMD, significant technological and physiological limitations remain. For one, communication in these paradigms is often limited to simple yes/no questions through mental-imagery tasks. Consequently, the nuanced discussions of risks, benefits, and alternatives required for informed consent are severely constrained by the fatigability and fluctuating attention characteristic for these patients. It is therefore likely that only a small subset of patients with DoC will be able to use these technologies to provide informed consent and engage in medical decision making. Regardless, though, there may be a significant therapeutic benefit to utilizing these technologies, as it may provide an “enormous opportunity to regain a sense of control within their medical treatment trajectory, which would contribute to their physical and mental wellbeing” (40). These technologies may give us a window into a patient's present state of mind and potential for suffering, which many surrogate decision makers have cited as an important factor in their substituted judgment (41, 42). Even if these technologies are not yet reliable enough for formal medical decision-making, the act of eliciting patients' thoughts may itself be valuable, and a mechanism for improving QoL for these patients in and of itself.

Much research to date on patients with CMD has focused on functional outcomes and prognostication rather than quality of life itself. Despite the clear positive correlation that CMD has with functional outcomes, unfortunately a substantial proportion of patients with CMD will not achieve the outcomes these technologies promise. Available evidence suggests that approximately 60% of patients with CMD will not recover to partial functional independence (defined as Glasgow Outcome Scale >/=4) (11), and 15%−30% of patients with CMD will not regain consciousness at all (43, 44). For these patients, using these neurotechnologies therapeutically—not merely diagnostically—could provide meaningful opportunities to express needs, whether they be social, emotional, or physical. It could also provide an opportunity to facilitate communication between loved ones, which could benefit families and patients alike. Such benefits would be fully realized even in the absence of favorable functional outcomes. While the technologies for detecting CMD have not been optimized or designed for this purpose, it would be a missed opportunity not to explore their utility in improving the lives of these patients during their long courses of neurorecovery.

## Conclusion

The discovery of CMD has rightly challenged the therapeutic nihilism that long defined the care of patients with disorders of consciousness, offering renewed hope for recovery and a more nuanced understanding of awareness after severe brain injury. Yet as these technologies reshape clinical practice, it is essential that they not simply replace one reductive framework with another—substituting the assumption that unresponsive patients lack consciousness with the assumption that consciousness can only be affirmed through neuroimaging. The possibility of covert consciousness in patients who cannot communicate presents a new source of clinical uncertainty in an already diagnostically challenging field, and the technologies themselves are not immune to this given their clear limitations. The stakes of diagnostic uncertainty here are uniquely high—they pertain not to inherently unknowable future outcomes, but to present, here-and-now experience that was previously inaccessible due to technological limitations. Further, if CMD reveals the possibility of awareness, then it also reveals the possibility of existential distress—of a conscious mind unable to act, communicate, or seek meaning—and this demands a palliative response that extends beyond analgesia to encompass the social, emotional, and spiritual dimensions of care. This revelation applies not only to patients who clearly demonstrate CMD, but potentially all patients with DoC. It is imperative to remain humble in the face of diagnostic uncertainty and avoid prematurely ruling out consciousness in patients who cannot meet the demands of CMD testing in its current iteration, as there may yet be opportunities for improving their QoL. These advanced neurotechnologies, used not only as diagnostic or prognostic tools, but as therapeutic modalities for communication and connection, may offer patients with CMD a measure of agency and self-expression that has value independent of any prognostic outcome. Ultimately, the ethical imperative in disorders of consciousness is to respect the experience of the mind. With the advent of advanced neurotechnologies, the obligation to attend to this experience with compassion and humility does not resolve, but deepens.

## Data Availability

The original contributions presented in the study are included in the article/supplementary material, further inquiries can be directed to the corresponding author.
